# Graphene-driving strain engineering to enable strain-free epitaxy of AlN film for deep ultraviolet light-emitting diode

**DOI:** 10.1038/s41377-022-00756-1

**Published:** 2022-04-07

**Authors:** Hongliang Chang, Zhetong Liu, Shenyuan Yang, Yaqi Gao, Jingyuan Shan, Bingyao Liu, Jingyu Sun, Zhaolong Chen, Jianchang Yan, Zhiqiang Liu, Junxi Wang, Peng Gao, Jinmin Li, Zhongfan Liu, Tongbo Wei

**Affiliations:** 1 Research and Development Center for Semiconductor Lighting Technology, Institute of Semiconductors, Chinese Academy of Sciences, 100083 Beijing, China; 2 Center of Materials Science and Optoelectronics Engineering, University of Chinese Academy of Sciences, 100049 Beijing, China; 3 Center for Nanochemistry (CNC), Beijing Science and Engineering Center for Nanocarbons, Beijing National Laboratory for Molecular Sciences, College of Chemistry and Molecular Engineering, Peking University, 100871 Beijing, China; 4 Electron Microscopy Laboratory, and International Center for Quantum Materials, School of Physics, Peking University, 100871 Beijing, China; 5 Beijing graphene institute (BGI), 100095 Beijing, China; 6 Academy for Advanced Interdisciplinary Studies, Interdisciplinary Institute of Light-Element Quantum Materials and Research Center for Light-Element Advanced Materials, Peking University, 100871 Beijing, China; 7 State Key Laboratory of Superlattices and Microstructures, Institute of Semiconductors, Chinese Academy of Sciences, 100083 Beijing, China

**Keywords:** Inorganic LEDs, Optoelectronic devices and components

## Abstract

The energy-efficient deep ultraviolet (DUV) optoelectronic devices suffer from critical issues associated with the poor quality and large strain of nitride material system caused by the inherent mismatch of heteroepitaxy. In this work, we have prepared the strain-free AlN film with low dislocation density (DD) by graphene (Gr)-driving strain-pre-store engineering and a unique mechanism of strain-relaxation in quasi-van der Waals (QvdW) epitaxy is presented. The DD in AlN epilayer with Gr exhibits an anomalous sawtooth-like evolution during the whole epitaxy process. Gr can help to enable the annihilation of the dislocations originated from the interface between AlN and Gr/sapphire by impelling a lateral two-dimensional growth mode. Remarkably, it can induce AlN epilayer to pre-store sufficient tensile strain during the early growth stage and thus compensate the compressive strain caused by hetero-mismatch. Therefore, the low-strain state of the DUV light-emitting diode (DUV-LED) epitaxial structure is realized on the strain-free AlN template with Gr. Furthermore, the DUV-LED with Gr demonstrate 2.1 times enhancement of light output power and a better stability of luminous wavelength compared to that on bare sapphire. An in-depth understanding of this work reveals diverse beneficial impacts of Gr on nitride growth and provides a novel strategy of relaxing the vital requirements of hetero-mismatch in conventional heteroepitaxy.

## Abstract (web summary)

This work successfully achieves a strain-free AlN film with low dislocation density for DUV-LED through graphene-driving strain-pre-store engineering and present the unique mechanism of strain-relaxation in QvdW epitaxy.

## Graphical abstract

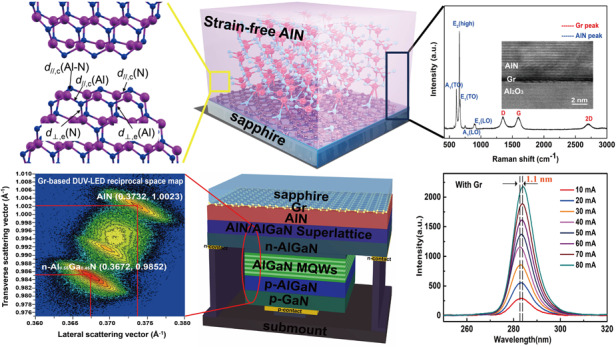

## Introduction

The direct band gap of AlN-based materials makes them suitable for fabricating optoelectronic devices^1–3^, especially for deep ultraviolet (DUV) wave band^4–7^, which have a wide range of application prospects in the fields of sterilization, polymer curing, biochemical detection, non-line-of-sight communication, and special lighting^8–10^. Therefore, achieving a high-quality epitaxy of AlN films is of particular importance to ensure the excellent performance of DUV photoelectric devices^11–13^. Currently, due to the lack of large-size and low-price homogenous substrates, the optimal choice to grow AlN films is usually to perform heteroepitaxial growth on sapphire^14–17^. Unfortunately, due to the inherent lattice and thermal expansion coefficient (TEC) mismatches between AlN and sapphire substrate^18,19^, even the well-known two-step epitaxy method or epitaxial lateral overgrowth (ELO) technology still inevitably introduces a variety of crystal defects into AlN epilayer^20–22^. In particular, the large residual strain in the AlN film leads to the nonuniformity of the Al distribution in the upper AlGaN layer accompanied by wafer bending, which severely limits the device performance^23,24^. The way to release large residual strain of the film is to mitigate the strong interactions between the substrate and the epilayer, or to introduce “negative strain” to neutralize the original residual strain^25,26^. Therefore, a more feasible strategy is straightaway required to make a qualitative leap to realize high-quality growth of heteroepitaxial AlN films and to meet the application requirements of DUV optoelectronic devices.

Over recent years, an emerging method named quasi-van der Waals (QvdW) epitaxy or remote epitaxy based on two-dimensional (2D) material has been proposed for high-quality heteroepitaxial growth of group-III nitrides^27–30^. The widely studied 2D material, graphene (Gr) has been incorporated as a buffer layer for the epitaxial growth of nitrides to effectively alleviate the lattice and TEC mismatches between the epilayer and the substrate^31–33^. Besides, it is worth mentioning that the metal adatoms of nitrides have a tiny migration barrier on the Gr surface, which can undoubtedly promote the 2D growth trend of nitride films^34,35^. Kim et al. demonstrated direct vdW epitaxy of high-quality single-crystalline GaN film on epitaxial Gr with low defects, and realized the epilayer transfer from Gr/SiC substrates^36^. Chung et al. achieved the growth of high-quality GaN films utilizing Gr/ZnO nanowalls as an interlayer and fabricated GaN-based blue LED that can be transferred to foreign substrates^37^. However, the previously reports on the epitaxial nitride film on Gr usually stated that the stress relaxation of epitaxial system is realized through the weak interaction between Gr itself and epilayers, but there is no detailed discussion or rigorous verification of this statement^32,38^. Until 2020, Bae et al. reported the discovery of a novel pathway of relaxing misfit strain in heteroepitaxial films via interface displacement on Gr-coated substrates^25^. The aforementioned material systems are those with relatively small lattice mismatch (InGaP on GaAs with 0.74% mismatch and GaP on GaAs with 3.7% mismatch) and the Gr undergoes a complicated transfer process instead of directly growing on the heterogeneous substrate. However, the potential of Gr film as an interlayer to release the strain in a large-mismatched epitaxial material system (i.e., AlN on c-sapphire with 13.3% lattice mismatch and 44% TEC mismatch) is still ambiguous and needs further development. Meanwhile, Dou et al. observed the chemical bond formation at the interface between the directly grown Gr and sapphire by aberration-corrected transmission electron microscopy (TEM) and found the strong interaction between Gr and sapphire^39^, which will inevitably change the interface displacement of Gr during QvdW growth. Therefore, there must be a novel QvdW epitaxial mechanism to explore for nitrides on the directly grown Gr-covered substrate. Unfortunately, there have been few reports to reveal the QvdW epitaxy mechanism of AlN films, which is essential to precisely manipulate the epitaxial quality of AlN films and further elevate the performance of DUV optoelectronic devices.

Herein, we successfully achieve a strain-free AlN film with low dislocation density (DD) through Gr-driving strain-pre-store engineering and present the unique mechanism of strain-relaxation in QvdW epitaxy, different from the previous “interface displacement” argument^25^. According to the results obtained by X-ray diffraction (XRD) and TEM, the DD of AlN with Gr exhibits an anomalous sawtooth-like evolution during the QvdW epitaxy process and the values are consistently lower than that without Gr. More importantly, combining Raman analysis and first-principles calculations, it is revealed that plasma-treated Gr with a high density of small-size AlN nucleation islands will pre-store sufficient tensile strain during the coalescence process. The tensile strain can compensate the compressive strain caused by lattice and TEC mismatches during heteroepitaxy, thus bringing out a strain-free AlN film. The reciprocal space mapping (RSM) of the as-fabricated DUV light-emitting diode (DUV-LED) reveals only a weak compressive strain in the n-AlGaN layer, enabling the high-quality crystalline state of the upper LED structure. The as-fabricated 283 nm DUV-LED with Gr demonstrates 2.1 times higher light output power (LOP) compared to its counterpart on bare sapphire and better stability of luminous wavelength under the changing injection current. This work reveals the internal mechanism of QvdW growth of AlN on large-mismatched substrate and undoubtedly sheds light on the further promotion of nitride-based device manufacturing.

## Results

**Fig. 1:**
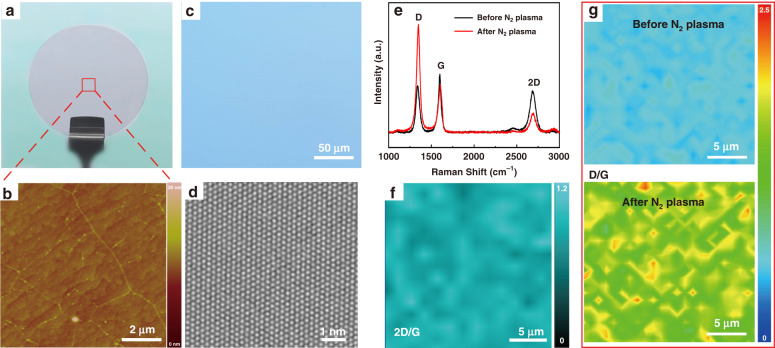
AFM and Raman analyses of Gr-covered sapphire substrate. **a** Photograph of an as-grown 2-inch Gr/sapphire wafer. **b** AFM image of the as-grown Gr on sapphire. **c** OM image of the as-grown Gr films after transferred onto SiO_2_/Si substrate. **d** Representative atomically resolved image of Gr. **e** Raman spectra of Gr on sapphire before (black) and after (red) N_2_ plasma. **f** *I*_2D_/*I*_G_ ratio for the Gr film in a 20 × 20 μm^2^ region. **g** Raman mapping of *I*_D_/*I*_G_ obtained from Gr film before (upper) and after (lower) N_2_ plasma treatment

To avoid repeatability limitation caused by the complicated transfer process, Gr with high scalability is directly grown on the c-plane (0001) sapphire via a catalyst-free atmosphere chemical vapor deposition (CVD) method at 1050 °C for 3 h, under a gas mixture of Ar, H_2_ and 30 sccm CH_4_^40^. Figure 1a shows a photograph of the as-grown 2-inch Gr/sapphire wafer, while the enlarged atomic force microscope (AFM) image (Fig. 1b) reveals the spotless Gr film with a flat surface and a crystal domain size of around 2–4 μm on sapphire. Through optical microscope (OM) inspection, the as-grown Gr film transferred to the SiO_2_/Si substrate exhibits the same color contrast that indicates its good uniformity (Fig. 1c). And the high quality of the Gr film grown on sapphire is confirmed by high-resolution TEM characterization in Fig. 1d. The typical Raman spectrum (black curve in Fig. 1e) as well as Raman mapping (Fig. S1a–c, Supporting Information) of the grown Gr show the characteristic Raman peaks of Gr at D-1341 cm^−1^, G-1585 cm^−1^, and 2D-2684 cm^−1^ with a high microscale uniformity. In particular, as shown in Raman mapping of Fig. 1f, the 2D/G intensity ratio is always greater than 1 at the micron-scale, so it can be considered that the Gr film is dominated by 1–2 layer^41^. Since it is difficult to grow AlN directly on the pristine Gr film, thus N_2_ plasma treatment has been employed to introduce defects into the Gr film to enhance its chemical reactivity before the AlN epitaxial growth^6^. It can be seen from Fig. 1e that there is a key difference between the Raman spectrum of the monolayer Gr before and after the plasma treatment, namely that the D peak becomes more towering after the treatment. The I_D_/I_G_ is significantly enhanced over a mapping area of 20 × 20 μm^2^ after N_2_ plasma treatment (Fig. 1g). The average defect density can be calculated from Eq. (1):

1 $$n_{{{\mathrm{D}}}} = \frac{{\left( {{{{\mathrm{1}}}}{{{\mathrm{.8}}}} \pm {{{\mathrm{0}}}}{{{\mathrm{.5}}}}} \right) \times {{{\mathrm{10}}}}^{{{{\mathrm{22}}}}}}}{{\lambda _{{{\mathrm{L}}}}^4}}\left( {\frac{{I_{\rm{D}}}}{{I_{\rm{G}}}}} \right)$$

where *n*_D_, *I*, and λ_L_ represent the defect density, the peak intensity, and the laser wavelength, respectively^42^. Compared to the pristine Gr, the average defect density in Gr is increased from *n*_D_ = 1.77 × 10^11^ cm^−2^ to *n*_D_ = 3.48 × 10^11 ^cm^−2^, attributing to the increase of dangling bonds during the plasma treatment^40^.

**Fig. 2:**
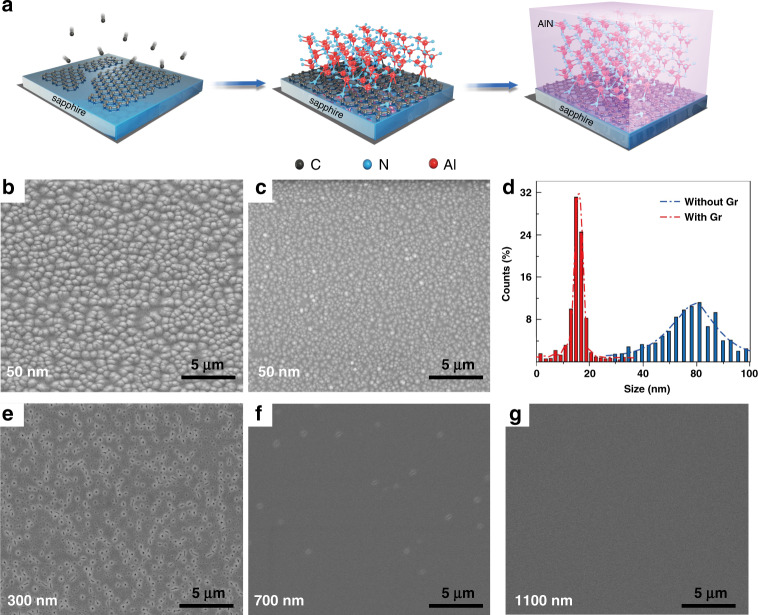
Morphology characterization of AlN nucleation and film growth on sapphire substrate with and without Gr buffer layer. **a** Schematic diagram of the key steps involved in the growth of high-quality AlN films on N_2_-plasma-treated Gr/sapphire substrate. **b**, **c** SEM images of AlN nucleation on the sapphire substrate (**b**) without and (**c**) with Gr. **d** Size distribution analysis of AlN nucleation on sapphire and Gr/sapphire substrate. **e**–**g** SEM images of AlN interrupted growth on Gr-buffered sapphire with thicknesses of (**e**) 300 nm, (**f**) 700 nm, and (**g**) 1100 nm

The growth interruption method is implemented to investigate the evolution of the morphology of AlN during the whole epitaxy process on sapphire with and without Gr. Firstly, the key processes involved in the growth of high-quality AlN films on the Gr layers are schematically shown in Fig. 2a. In brief, after the Gr film is grown directly on the sapphire, the defects are introduced into the Gr film by N_2_ plasma treatment, and then the epitaxy of the AlN film is performed. The nucleation of AlN mainly occurs at the N defect sites through Al–N bond as the fulcrum, and then AlN clusters eventually rapidly expand into the film in the form of vdW epitaxy driven by Gr^6^. For the growth on bare sapphire, the AlN epilayer is initiated with the thin nucleation at high temperature (Fig. 2b). Here, according to the scanning electron microscope (SEM) image in Fig. 2b and statistics in Fig. 2d, the hexagonal AlN nucleation islands grown on the bare sapphire are quite large and widely distributed with an average diameter and an island density of 84 nm and 4.2 × 10^9^/cm^2^, respectively. As the growth thickness increases, the AlN nucleation islands on the bare sapphire do not tend to coalescent with each other. They rather expand with a typical 3D-dominated Volmer–Weber growth mode, thus resulting in the rough surface (Fig. S2, Supporting Information). On the contrary, the introduction of Gr significantly changes the above-mentioned growth mode. In the initial stage of growth, higher-density and smaller-size nucleation islands have been formed on the Gr-buffered sapphire (Fig. 2c). From Fig. 2c, d, it can be estimated that the AlN nucleation islands reach a density of 1.28 × 10^11^/cm^2^ and an average diameter of 17 nm (only about one-fifth of AlN nucleation islands on bare sapphire). It is attributed to the adoption of N_2_ plasma treatment prior to growth, which can greatly facilitate AlN nucleation by introducing pyrrolic-nitrogen defects into Gr^6^. Furthermore, since Al atoms have an extremely low migration barrier (less than 0.1 eV) on Gr^34^, the nucleated AlN rapidly coalesces to fully cover the Gr-buffered sapphire (Fig. 2e). A quasi-flat surface morphology with sporadic micro-pits is formed at a thickness of only 700 nm (Fig. 2f), while the mirror-smooth surface with a root mean square (RMS) roughness of only 0.256 nm (Fig. S3, Supporting Information) is finally realized with a thickness of 1100 nm in Fig. 2g.

**Fig. 3:**
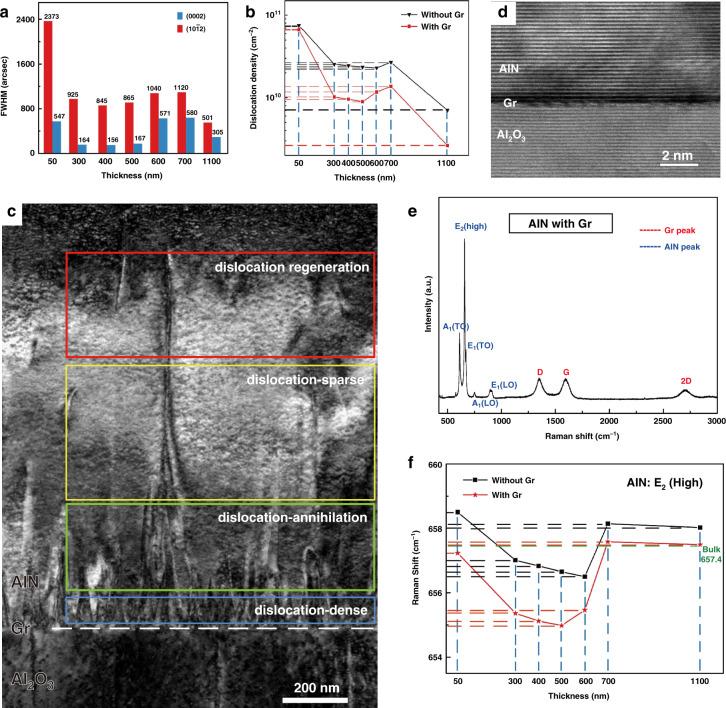
XRC, TEM and Raman analyses for crystal quality of as-grown AlN film. **a** FWHMs of (0002)- and (10$\bar 1$2)-plane XRCs of AlN epilayer with various thicknesses grown on Gr-buffered sapphire. **b** Estimated DD of the AlN films with and without Gr buffer layer with various thicknesses. **c** DF images of epitaxial AlN/Gr/sapphire with g = [0002]. **d** HRTEM image of the AlN/Gr/sapphire interface. **e** Raman spectra of as-grown AlN/Gr/sapphire structure. **f** Relative Raman shifts of E_2_ (high) of AlN with various growth thicknesses

The crystallinity of the as-grown epilayers with various thicknesses is reflected by the full width at half maximum (FWHM) of the X-ray rocking curve (XRC) of AlN film^43^. From the histogram in Fig. 3a, it is noted that the FWHM value of AlN films with Gr shows a nonmonotonic variation during the film growth process, instead of a simple decreasing with the increase of film thickness, regardless of (0002) and (10$\bar 1$2) planes. For the seven thicknesses of the epilayers, the DD estimated from the XRC FWHM illustrates an anomalous sawtooth-like evolution for both AlN films with (red broken line in Fig. 3b) and without Gr (black broken line in Fig. 3b). It confirms the secondary increase stage of the DD of AlN epilayer on Gr during the growth stage from 500 to 700 nm, while that on sapphire is from 600 to 700 nm^44^. The specific values of the DD of AlN films with and without Gr are listed in Table S1 (Supporting Information). It can be found that the DD of the AlN epilayers with Gr is indeed lower than those of AlN epilayer grown on bare sapphire during the whole epitaxy process. Finally, the introduction of Gr leads to a decrease of the DD of the AlN film by 62.6% (Table S1, Supporting Information). The selected-area electron diffraction (SAED) pattern from the interface region certifies the orientation relationships of (0002) AlN // (0006) Al_2_O_3_ and (1$\bar 1$00) AlN // (11$\bar 2$0) Al_2_O_3_ (Fig. S4, Supporting Information). Meanwhile, a dark field (DF) cross-sectional TEM image of AlN grown on Gr-buffered sapphire with *g* = [0002] is shown in Fig. 3c. Obviously, the threading dislocations originate from the interface between AlN and Gr/sapphire and first form the dislocation-dense region, followed by sharp bending and annihilation within a thickness of approximately 300 nm (dislocation-annihilation region). The partial propagating termination of dislocations in the upper epilayer may be attributed to the lateral 2D growth mode actuated by Gr. After the dislocation-sparse region of about 400 nm, some new dislocations emerge at a distance of about 600–700 nm from the AlN/Gr interface inside the epilayer, consistent with the aforementioned results of the secondary increase stage determined by XRC. The high-resolution transmission electron microscopy (HRTEM) image of AlN/Gr/sapphire interface shows the existence of an approximately 0.7-nm-thick Gr layer (dark area in Fig. 3d). Raman spectra also show signal peaks of both AlN and Gr, indicating Gr keeps its original layered structure during AlN growth at high temperature (Fig. 3d and Fig. S5, Supporting Information). Furthermore, from the perspective of the epitaxial mechanism, the stable existence of Gr provides an important prerequisite for its continuous function in the nucleation, diffusion, and coalescence of AlN, thus ensuring the established growth mode of QvdW epitaxy.

In order to understand the stress relaxation of AlN films grown on sapphire with and without Gr during the heteroepitaxy, we explore the strain state of AlN epilayer with different growth thicknesses by characterizing the stress-sensitive E_2_ (high) phonon mode in the Raman spectrum, as depicted in Fig. 3e and Table S2 (Supporting Information)^45^. In the initial growth stage, regardless of the presence of Gr, the position of E_2_ (high) peak blue-shifts continuously. For the AlN epilayer about 700 nm on sapphire, the position of E_2_ (high) peak suddenly red-shifts. In contrast, the tensile strain accumulation of the AlN epilayer on Gr is more intense in the initial stage, so the turning point of E_2_ (high) peak of the AlN epilayer on Gr appears earlier at 600-nm thickness, followed by a similar sharp increase at 700 nm. When the position of E_2_ (high) peak shifts to a higher wavenumber for 700-nm-thick AlN epilayer, the strain state of the epilayer has changed from tensile to compressive strain. Therefore, it is reasonable to speculate that the regeneration of the dislocations at a thickness of about 600–700 nm observed in the TEM originates from the release of the accumulated tensile strain in the epilayer. The residual compressive strain should be attributed to the TEC mismatch during the cooling process. More importantly, from a macro perspective, the wavenumber of the E_2_ peak of AlN epilayer with Gr is always smaller than that of AlN on bare sapphire under the same thickness, suggesting that the presence of Gr provides an additional source of tensile strain in the epitaxy system. Consequently, the strain-free AlN film based on Gr is eventually obtained, while AlN film on bare sapphire demonstrates the residual compressive strain. In addition, RSM by high-resolution XRD is ideally suited to detailed structural characterization of layered nitride structure. As shown in Fig. S6 (Supporting Information), the reciprocal space coordinate position (RSCP) corresponding to the (10$\bar 1$5) crystal plane of the AlN epilayer is almost identical to that of the intrinsic bulk-AlN (0.3711, 1.0036), reflecting the perfect lattice state without the residual strain of AlN film grown on Gr^46^.

**Fig. 4:**
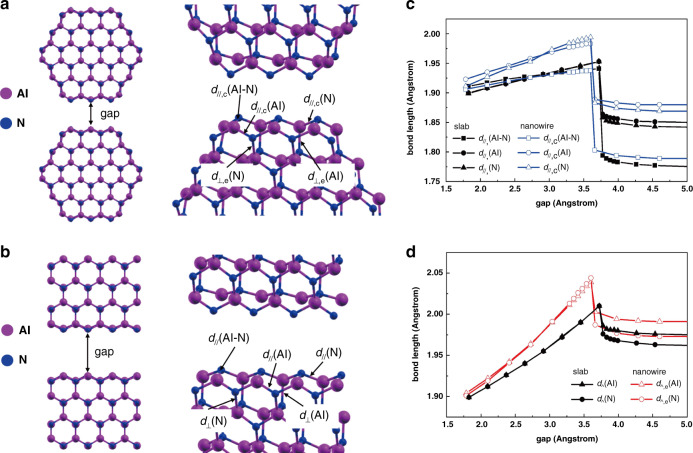
DFT calculations of the coalescence between two AlN (1$\bar 1$00) surfaces. **a** Schematic plot of the coalescence between two AlN nanowires and the surface bond lengths. **b** Schematic plot of the coalescence between two infinite AlN surfaces and the surface bond lengths. *d*_//_(Al-N) is the bond length of the Al-N bond formed by the surface Al and N atoms parallel to the surface, *d*_//_(Al) is the bond length of the parallel Al-N bond formed by the surface Al and subsurface N atom, an*d d*_//_(N) is the bond length of the parallel Al-N bond formed by the surface N and subsurface Al atom. The notions for perpendicular bonds are similar. The subscripts e and c denote the bonds of the nanowire on the edge and near the corner, respectively. **c** The variation of the parallel bond lengths of nanowire and infinite surface as a function of the separation gap. **d** The variation of the perpendicular bond lengths of nanowire and infinite surfaces as a function of the separation gap

As a matter of fact, the key to growing high-quality strain-free film is to balance the competitive relationship between the inherent compressive strain and the tensile strain in the epilayer. From the previous studies^2,47^, it is determined that the positive lattice mismatch of +13.3% and the negative TEC mismatch of −44% inevitably exist between AlN and c-surface sapphire, which enforces the AlN epilayer grown on sapphire to exhibit a compressive strain. On the other hand, there exists a source of tensile stress during the coalescence process. As the atoms on the crystalline planes from different nuclear islands start to form chemical bonds with each other, the contact and coalescence of adjacent AlN clusters will inevitably induce the tensile strain. And the magnitude of the local tensile strain introduced in the coalescence process depends on the size of the nuclear islands that coalesce with each other^48^. Hence, in order to investigate the characteristics of the tensile strain between adjacent AlN clusters developed during the coalescence process, we performed first-principles calculations based on density functional theory (DFT) to study the structural relaxation of two surfaces at different separations. The most stable side facet of the AlN clusters is the (1$\bar 1$00) surface. Based on the assumption that the surface structural relaxation depends on the size of the AlN clusters, we calculate two extreme cases: one is the coalescence of two AlN nanowires with diameters of 15.6 Å, and the other is the coalescence of two infinite AlN (1$\bar 1$00) surfaces of an AlN slab, as shown in Fig. 4a, b. Here, we focus on the tensile strain due to the outwards relaxation of the surface atoms rather than the shifts of the whole cluster, and thus keep the centers of the AlN nanowires and slabs fixed. We gradually change the gap i.e., the distance between the two surfaces before relaxation and then relax the structures. The surface bonds are measured to analyze the strain at different gaps.

We first consider the bonds parallel to the coalescent surfaces. At a larger gap, these surface bonds are always shorter than those in bulk due to surface contraction, and slightly elongate when the gap decreases, as shown in Fig. 4c. At a critical gap, the atoms on the two separated surfaces form chemical bonds with each other, and the lengths of these surface bonds increase dramatically to a value larger than the bulk bond length, and then gradually shorten to bulk value until the gap decreases to the bulk gap at 1.8 Å. The critical gaps for a small nanowire and infinite surface are quite similar around 3.6 Å. Between the critical gap and the bulk gap, the elongation of these surface bonds induces a tensile strain to the AlN clusters. For example, at the critical gap between infinite surfaces, the surface-parallel Al–N bond length is *d*_//_(Al–N) =1.94 Å, 0.3 Å longer than the bond length in the bulk.

In nanowires, the surface bonds at the edge and near the corner exhibit different variations as the gap decreases. The elongation of the surface-parallel bonds on the edge of the nanowire is similar to those on the infinite surface (Fig. S7b, Supporting Information). However, the elongation of the surface bonds near the corner of the nanowire is larger than that on the infinite surface. As shown in Fig. 4c, the bonds near the corner of the small nanowire (*d*_//,c_(Al) and *d*_//,c_(N)) are longer than those of infinite surface by about 0.04 Å at the critical gap of 3.6 Å. This indicates that the tensile strain of small AlN clusters is larger than that of large clusters during the coalescence. The larger elongation of the corner bonds on nanowires is due to a higher degree of freedom of the corner atoms. Compared to the atoms on the infinite surface, the corner atoms of a nanowire can move outwards by a larger distance.

Similarly, we measure the bonds perpendicular to the coalescent surface. Due to the surface in-plane contraction, the perpendicular bonds on both nanowire and infinite surface are longer than the bulk bonds. As shown in Fig. 4d, the lengths of the perpendicular bonds also experience a dramatic increase at the critical gap. Furthermore, the perpendicular bonds on the edge of the nanowire (*d*_⊥,e_(Al) and *d*_⊥,e_(N)) are elongated by a larger amount than those on the infinite surface. The perpendicular bonds on the corner of the nanowire are essentially the surface bonds on the neighboring facets and thus have a smaller elongation (Fig. S7c, Supporting Information). According to the above calculation results, we can conclude that two AlN clusters will coalesce into a large one when the separation between them is smaller than the critical gap. During the coalescence process, the surface atoms will shift outwards forming chemical bonds with the other surface. For a smaller AlN cluster, the surface atoms can move outwards by a larger distance. And the parallel bonds near the corner and the perpendicular bonds on the edge will experience a larger elongation than those of a larger cluster. As a result, a smaller AlN cluster will experience a greater tensile strain than a larger cluster during the coalescence process.

**Fig. 5:**
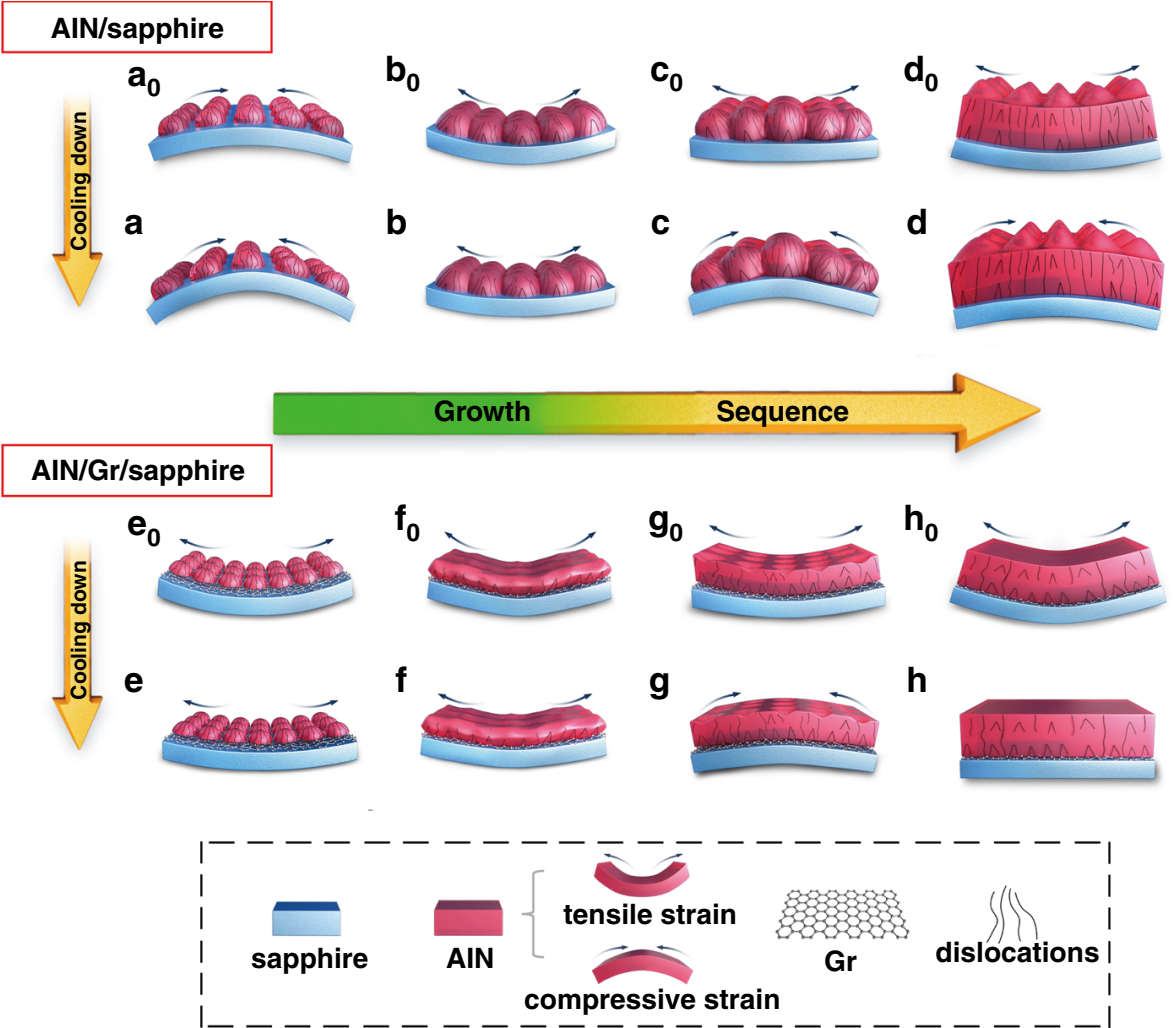
Schematic diagrams of the epitaxial process of AlN film grown on Gr/Sapphire. The growth development of AlN films without [(**a**_**0**_)–(**d**_**0**_) and (**a**)–(**d**)] and with Gr [(**e**_**0**_)–(**h**_**0**_) and (**e**)–(**h**)] during the processes from nucleation growth [(**a**_**0**_)–(**a**) and (**e**_**0**_)–(**e**)] to nucleation island coalescence [(**b**_**0**_)–(**b**) and (**f**_**0**_)–(**f**)] to completion of coalescence [(**c**_**0**_)–(**c**) and (**g**_**0**_)–(**g**)] and final film formation [(**d**_**0**_)–(**d**) and (**h**_**0**_)–(**h**)]. All the pictures with “0” subscripts present the state of processes under high-temperature growth (not cooled to RT) and the images without “0” subscripts show the state after cooling down to room temperature

Based on the characterization analysis and DFT calculations mentioned above, a schematic growth model is proposed to elucidate the internal mechanism of Gr to affect the crystallinity and strain state of the AlN epilayer. As mentioned above, negative thermal mismatch will introduce compressive strain into the AlN epilayer. Therefore, in order to interpret the results of the Raman spectrum measured at room temperature (RT) more straightforwardly, the strain state of the epilayer at each stage from the cooling down to the growth interruption is also depicted. As shown in Fig. 5a_0_, in the initial growth stage, the AlN nuclear islands grown on bare sapphire undergo a compressive strain due to the positive lattice mismatch, which is strengthened during the cooling process after growth due to TEC mismatch (Fig. 5a). Meanwhile, due to the existence of lattice mismatch, a large number of dislocations are produced at the AlN/sapphire hetero-interface and propagate upward. Subsequently, when the nucleation islands gradually coalesce, the DD decreases and the epilayer enters into a tensile strain state (Fig. 5b_0_), which becomes more and more stronger as the epilayer grows thicker. To a certain extent, the tensile strain dominates the AlN epilayer even after cooling down to RT (Fig. 5b). After the accumulated tensile strain reaches a critical value, it can be released by generating crystal defects such as dislocations. Therefore, the evolution of DD during the growth process shows a phenomenon of secondary generation to release strain. The remaining residual tensile stress will be compensated by the compressive strain produced due to hereto-mismatch (Fig. 5c_0_). However, although the state of tensile strain is maintained until the AlN film is completely formed at high temperature (Fig. 5d_0_), the AlN epilayer grown on sapphire eventually exhibits compressive stress due to thermal mismatch during the cooling process as shown in Fig. 5c, d. It is also worth noting that in the final film formation process from Fig. 5c_0_ to Fig. 5d_0_, the coalescence of the crystal columns will likewise introduce weak tensile strain^24^.

Providentially, the Gr interlayer can effectively improve the strain compensation. As an interlayer, Gr has a certain shielding effect on the heterogeneous substrate, which effectively reduces the number of dislocations generated at the AlN/sapphire hetero-interface (Fig. 5e_0_). Meanwhile, during the initial growth stage, the strong 2D growth mode driven by Gr promotes mutual annihilation of dislocations in the epilayer without extending upward to the surface of film (Fig. 5e). More importantly, as drawn in Fig. 5e_0_, f_0_ and e, f, the AlN epilayer demonstrates strong tensile strain at the initial epitaxy stage and even after cooling down to RT. In fact, N_2_ plasma-treated Gr promotes the formation of high-density and small-size nucleation islands of AlN. On one word, more coalescence interfaces between islands will undoubtedly induce larger tensile strain compared to its counterpart on bare sapphire. On the other word, according to first-principles calculation results, smaller-size AlN nucleation islands can also trigger more intense tensile strain during the coalescence process. As a result, even after partial strain release by the formation of dislocations, the AlN epilayer still suffers from a large tensile strain (Fig. 5g_0_), enough to compensate most of the compressive stress caused by TEC mismatch during cooling down (Fig. 5g). Finally, as the film surface completely turns smooth, a stress-free high-quality AlN film is obtained (Fig. 5h). Overall, large pre-stored tensile strain induced by Gr during the coalescence provides the guarantee to realize the strain-free AlN film. It can be further modulated by adjusting the nucleation density of QvdW growth.

**Fig. 6:**
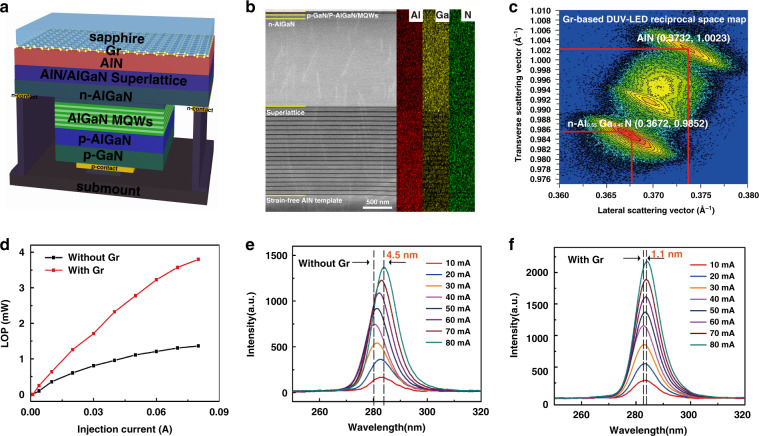
The structural characterization and EL properties of as-fabricated DUV-LEDs. **a** Schematic diagram of the DUV-LED structure with Gr. **b** Cross-sectional STEM image of as-grown DUV-LED with Gr and corresponding EDS mapping images of Al, Ga and N elements. **c** RSM for the (10$\bar 1$5) reflection of the DUV-LED grown on Gr/sapphire. **d** LOP of the as-fabricated DUV-LEDs with and without Gr as a function of the injection current. **e** EL spectra of DUV-LED without Gr by varying the injection current from 10 to 80 mA. **f** EL spectra of DUV-LED with Gr by varying the injection current from 10 to 80 mA

Finally, an AlGaN-based DUV-LED device is fabricated on the as-grown AlN/Gr/sapphire template, as schematically shown in Fig. 6a. From the SEM and AFM images, it can be seen that the surface of the as-grown Gr/sapphire-based DUV-LED is continuous and smooth with an RMS roughness of ~0.592 nm (Fig. S8, Supporting Information). Figure 6b and Fig. S9 (Supporting Information) show the low magnification cross-sectional scanning TEM (STEM) image of the DUV-LED grown on the AlN/Gr/sapphire template and corresponding energy dispersive spectroscopy (EDS) mapping images, which clearly show the DUV-LED heterojunction structure. As is shown in Gr-based DUV-LED RSM of Fig. 6c, the scattering corresponding to AlGaN layers is below the stronger AlN peak. And the RSCP corresponding to the (10$\bar 1$5) crystal plane of the 1.8 μm n-Al_0.55_Ga_0.45_N layer (0.3672, 0.9852) with AlN/Gr shows a tiny deviation from the intrinsic AlGaN (0.3669, 0.9855) with the Al composition of 0.55, suggesting that there is only a weak biaxial compressive strain in n-AlGaN layer^49^. However, the n-Al_0.55_Ga_0.45_N layer (0.3683, 0.9840) contained in the DUV-LED on bare sapphire shows the existence of greater compressive strain (Fig. S9, Supporting Information). The strain-free AlN film on Gr/sapphire can serve as a reliable template layer for the high-quality epitaxy of the LED structure and provides material guarantee for the fabrication of the LED devices. Moreover, after the epitaxial LED structure, the previous strain-free AlN film with Gr shows slight residual compressive strain, which may be attributed to the re-introduced strain during cooling down at the end of the LED epitaxy process. In order to evaluate the optical and electric properties of the as-fabricated DUV-LEDs, a series of electroluminescence (EL) properties of the devices with and without Gr are further studied. As plotted in Fig. 6d, the LOP of the LED with Gr increases linearly with increasing the injection current and exhibits a steeper slope efficiency of ~60 μW mA^−1^ than that on bare sapphire, suggesting that the EL emission is generated from the carrier injection and radiative recombination at the multiple quantum wells (MQWs) layers, thus ensuring its high internal quantum efficiency (IQE). At 20 mA, the LOP of the DUV-LED with Gr reaches 1.26 mW, which is 2.1 times higher than that without Gr (0.60 mW), confirming the high quality of the DUV-LED device due to the Gr buffer layer. According to the calculation, when the applied current is 20 mA, the corresponding external quantum efficiency (EQE) and wall-plug efficiency (WPE) of DUV-LED with Gr buffer layer are 1.44% and 1.02%, respectively, which are both higher than the EQE (0.68%) and WPE (0.5%) of LED without Gr. Moreover, the current-voltage curve of the DUV-LED with Gr exhibits a good rectifying behavior with a turn-on voltage of ~4.6 V (Fig. S11a, Supporting Information) and a low leakage current of 24 nA at −8 V (Fig. S11b, Supporting Information). As shown in Fig. 6d, e, the luminescence peak of DUV-LED on Gr/sapphire is located around 283 nm. With the injected current increasing from 10 mA to 80 mA, there is a 4.5 nm shift for the EL peak wavelength of DUV-LED on bare sapphire. In comparison, the DUV-LED with Gr exhibits a tiny wavelength-shift of 1.1 nm, including a blue-shift of only 0.4 nm, which shows negligible sensitivity to the current. It is attributed to the better crystalline quality with a weak residual strain of the epitaxial structure based on the Gr layer, which effectively suppresses the quantum confined Stark effect (QCSE) effect caused by the formation of spontaneous polarization field and reduces the non-radiative recombination normally associated with device heating^50,51^. Hence, this work illustrates that high-quality AlN achieved with the aid of the Gr enables the manufacture of high-performance DUV-LED.

## Discussion

Since the inherent lattice and TEC mismatch between AlN and sapphire substrate (a positive lattice mismatch of +13.3% and a negative TEC mismatch of −44%)^18,19^, crystal defects will inevitably be introduced into the AlN layer in the epitaxial process^20–22^ and the formed large residual strain severely limits the performance of the device^23^. Therefore, an excellent solution is needed to release the large residual strain of the epilayer, so as to realize the high-quality growth of the heteroepitaxial AlN film and meet the application requirements of DUV optoelectronic devices. With the assist of Gr-driving strain-pre-store engineering, we obtain strain-free AlN film with low DD and propose a unique strain-relaxation mechanism for QvdW epitaxial nitrides that is different from the previous “interface displacement”^25^. It is observed through XRD and TEM measurements that the DDs of the AlN epilayer grown on Gr undergo an abnormal sawtooth-like evolution during the epitaxy. Combining Raman spectrum and DFT calculation, it is revealed that the small-size AlN nucleation islands induced by N_2_ plasma-treated Gr may pre-store enough high tensile strain in epilayer during the coalescence, which compensates the compressive strain caused by hetero-mismatch and enables the epitaxy growth of strain-free AlN films. Further, the fact that there is only weak compressive strain in the n-AlGaN layer characterized by RSM confirms the good crystalline state of the upper LED structure based on the high-quality AlN/Gr template. The as-fabricated 283 nm DUV-LED based on AlN/Gr/sapphire exhibits excellent photoelectric properties, with the LOP enhancement of 2.1 times compared to its counterpart on AlN/sapphire and a better stability of luminous wavelength with changing current.

In summary, through both experimental and theoretical analysis of DFT calculation, this work reveals the unique mechanism of strain-relaxation of AlN epilayer in QvdW epitaxy guided by Gr-driving strain-pre-store engineering. Furthermore, the epitaxial LED structure on the strain-free AlN also exhibits a low residual strain, and the DUV-LED based on strain-free AlN/Gr shows more excellent and more stable optoelectronic properties than that on bare sapphire, demonstrating the great application of the strain-free AlN film to high-performance DUV-LED. These valuable perceptions about the inherent potence of Gr related to the growth of nitride film undoubtedly provide fulfilling enlightenment for developing the practical application of Gr in the cutting-edge manufacturing of nitride-based optoelectronic devices.

## Materials and methods

### CVD Growth of Gr on sapphire

Typically, a two-inch sapphire substrate is cleaned with deionized water, ethanol, and acetone and then loaded into a three-zone high-temperature furnace. The furnace is heated to 1050 °C and stabilized for about 10 min under 500 sccm Ar and 300 sccm H_2_. Then 30 sccm CH_4_ is introduced into the reaction chamber as a carbon source for the growth of Gr on the sapphire substrate for about 3–5 h.

### MOCVD Growth of AlN on Sapphire with Gr buffer layer

The Gr/sapphire is exposed to N_2_ plasma treatment (PVA TePla AG, 300 Standard) under optimized plasma treatment conditions (200 Pa pressure with 300 sccm air flow and 50 w power for 30 s) before loading into MOCVD chamber. And the type of plasma treatment is reactive ion etching (RIE) which is capacitively coupled radio frequency (RF) plasma. Table S3 (Supporting Information) describes the process of selecting the optimal power intensity for plasma treatment applied to Gr in detail. Subsequently, the AlN films are simultaneously grown at 1270 °C for 2 h with an NH_3_ flow of 500 sccm and a trimethylaluminum (TMAl) flow of 70 sccm on sapphire with and without the Gr interlayer in a single experiment. And the V/III ratio of the subsequent growth process is 270. It is a one-step process without using LT AlN buffer layer.

### MOCVD growth of DUV-LED structure on Gr/sapphire

The MOCVD system used in the epitaxial growth process of DUV-LEDs is a home-made vertical system. The AlGaN-based DUV-LED structure is grown on the AlN/Gr/sapphire template, including a 20-period AlN/Al_0.6_Ga_0.4_N superlattice (SL), n-Al_0.55_Ga_0.45_N layer, five-period Al_0.4_Ga_0.6_N/Al_0.5_Ga_0.5_N MQWs, and p-type layers (layer of Mg-doped p-Al_0.65_Ga_0.35_N electron blocking layer (EBL), p-Al_0.5_Ga_0.5_N cladding layer and p-GaN contact layer). Trimethylgallium (TMGa) is used as a Ga precursor. Silane (SiH_4_) and bis (cyclopentadienyl) magnesium (Cp_2_Mg) are used for n-type and p-type doping, respectively. A 20-period AlN (60 nm)/Al_0.6_Ga_0.4_N (15 nm) SL is first deposited at 1130 °C, with the periodic flow change of TMAl to adjust the deposition component while the TMGa flow is kept at 32 sccm. Then temperature is reduced to 1002 °C, and 20 sccm silicane hydrogen mixture flow (actual silicane flow rate is 2.34 sccm) is introduced for the growth of the 1.8 μm n-Al_0.55_Ga_0.45_N layer. The five-period Al_0.5_Ga_0.5_N/Al_0.4_Ga_0.6_N MQWs structure is further grown with a 3 nm quantum well and a 12 nm quantum barrier by switching the TMAl from 24 to 14 sccm and TMGa from 8 to 7 sccm for each period. A 50 nm thick layer of Mg-doped p-Al_0.65_Ga_0.35_N EBL, a p-Al_0.5_Ga_0.5_N (30 nm) cladding layer, and a 100 nm thick p-GaN contact layer are subsequently deposited. After the growth, the p-type layers are annealed in the reactor at 800 °C in N_2_ atmosphere for 20 min to activate the Mg acceptors. In addition, the doping concentration of Si in the n-Al_0.55_Ga_0.45_N layer is 3 × 10^18^ cm^−3^ and the doping concentration of Mg in p-GaN is 1 × 10^18^ cm^−3^. Moreover, the corresponding estimated carrier concentrations of n-type layer and p-type layer are 3 × 10^18^ and 1 × 10^16^ cm^−3^, respectively.

### DUV-LED device fabrication

DUV-LED devices with a die size of 0.5 mm × 0.5 mm are fabricated following the standard LED processes. It includes the following steps: 1. Mesa etching: firstly, the positive-photoresist is used for photolithography of mesa pattern. Then, the photolithography pattern is further ICP etched with BCl_3_/Cl_2_/Ar mixed gas, and the etching depth was about 600 nm. 2. Fabrication of N electrode and p electrode: a combination method of negative-photoresist photolithography and metal stripping is adopted. By means of electron beam evaporation, the Ti/Al/Ti/Au metal stack is deposited on the exposed n-AlGaN as the n-type contact, while the Ni/Au stack is used as the p-type contact. 3. Passivation and isolation: SiO_2_ as isolation layer is deposited by PECVD to reduce leakage and avoid short circuit. Finally, the DUV-LED chips are flip-chip bonded onto ceramic submounts coated with gold for light output testing.

### Electron microscopy characterizations and image analysis

The cross-sectional TEM specimen is prepared by the focused ion beam system (ThermoFisher Helios G4 UX). The HRTEM images, DF images, and SAED patterns are performed on FEI Tecnai F20 TEM operated at 200 kV. And the HAADF images and EDS mapping are acquired at a spherical aberration-corrected FEI electron microscope (Titan Cubed Themis G2 300) operated at 300 kV. The camera length in HAADF mode is set as 145 mm. The convergence semi-angle of HAADF is 30 mrad and the collection semi-angle of HAADF is 39–200 mrad.

### Computational Method

The first-principles calculations are carried out using the Vienna ab-initio simulation package (VASP)^52^. We adopted the projector-augmented wave potentials and the generalized-gradient approximation of Perdew, Burke, and Ernzerhof for the exchange-correlation functional^53,54^. The coalescence between small clusters is modeled by two AlN nanowires with (1$\bar 1$00) facets. The cross section of the nanowire has three atomic shells. The coalescence between two infinite surfaces is modeled by an AlN (1$\bar 1$00) slab with 8 AlN bilayers. The inner shell of the nanowires and the centered two bilayers of the slab are kept fixed during structural relaxation to model the coalescence of AlN clusters without in-plane shifting on the substrates.

## Supplementary information

Supplementary Information For Graphene-driving strain engineering to enable strain-free epitaxy of AlN film for deep ultraviolet light-emitting diode

## Data Availability

All data supporting the findings of this study are available within the paper and its Supplementary Information
