# Author Correction: Graphene-driving strain engineering to enable strain-free epitaxy of AlN film for deep ultraviolet light-emitting diode

**DOI:** 10.1038/s41377-022-00802-y

**Published:** 2022-04-29

**Authors:** Hongliang Chang, Zhetong Liu, Shenyuan Yang, Yaqi Gao, Jingyuan Shan, Bingyao Liu, Jingyu Sun, Zhaolong Chen, Jianchang Yan, Zhiqiang Liu, Junxi Wang, Peng Gao, Jinmin Li, Zhongfan Liu, Tongbo Wei

**Affiliations:** 1 Research and Development Center for Semiconductor Lighting Technology, Institute of Semiconductors, Chinese Academy of Sciences, 100083 Beijing, China; 2 Center of Materials Science and Optoelectronics Engineering, University of Chinese Academy of Sciences, 100049 Beijing, China; 3 Center for Nanochemistry (CNC), Beijing Science and Engineering Center for Nanocarbons, Beijing National Laboratory for Molecular Sciences, College of Chemistry and Molecular Engineering, Peking University, 100871 Beijing, China; 4 Electron Microscopy Laboratory, and International Center for Quantum Materials, School of Physics, Peking University, 100871 Beijing, China; 5 Beijing graphene institute (BGI), 100095 Beijing, China; 6 Academy for Advanced Interdisciplinary Studies, Interdisciplinary Institute of Light-Element Quantum Materials and Research Center for Light-Element Advanced Materials, Peking University, 100871 Beijing, China; 7 State Key Laboratory of Superlattices and Microstructures, Institute of Semiconductors, Chinese Academy of Sciences, 100083 Beijing, China

**Keywords:** Inorganic LEDs, Optoelectronic devices and components

Correction to: *Light Science & Applications*

10.1038/s41377-022-00756-1 published online 07 April 2022

Following publication of this article^1^, it is noticed Fig. 6d contained a typo: the unit of x-coordinate should be A, not mA.

The correct Fig. 6d is provided in this Correction.
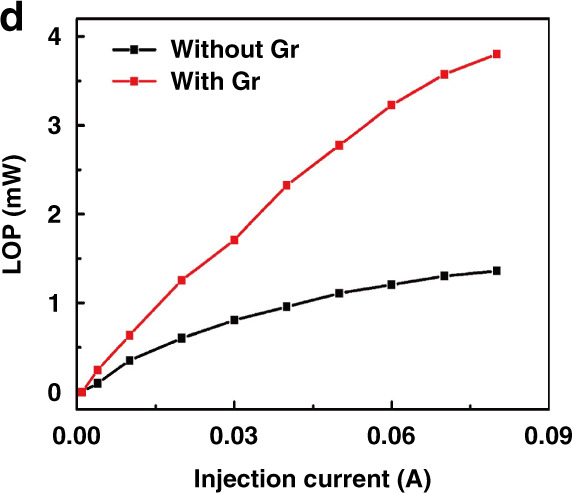


The original article has been updated.
