# Supplementary material for: Graphene-driving strain engineering to enable strain-free epitaxy of AlN film for deep ultraviolet light-emitting diode
Source: Light Sci Appl. 2022 Apr 7;11:88. doi: 10.1038/s41377-022-00756-1 (PMC8991230; doi:10.1038/s41377-022-00756-1)
Supplement: Supplementary file 1 — Supplementary Information For Graphene-driving strain engineering to enable strain-free epitaxy of AlN film for deep ultraviolet light-emitting diode [file 41377_2022_756_MOESM1_ESM.pdf]

Supplementary Information For

Graphene-driving strain engineering to enable strain-free epitaxy of AlN film for deep ultraviolet light-emitting diode

Hongliang Chang,[1,2#] Zhetong Liu,[3,4,5,6 #] Shenyuan Yang,[2,7#] Yaqi Gao,[1,2] Jingyuan Shan,[3,5] Bingyao Liu,[3,4,5,6] Jingyu Sun,[5] Zhaolong Chen,[3,5] Jianchang Yan,[1,2] Zhiqiang Liu,[1,2] Junxi Wang,[1,2] Peng Gao,[3,4,5,6]* Jinmin Li,[1,2]* Zhongfan Liu,[3,5]* Tongbo Wei[1,2]*

[1]Research and Development Center for Semiconductor Lighting Technology, Institute of Semiconductors, Chinese Academy of Sciences, Beijing, 100083, China

[2]Center of Materials Science and Optoelectronics Engineering, University of Chinese Academy of Sciences, Beijing 100049, China

[3]Center for Nanochemistry (CNC), Beijing Science and Engineering Center for Nanocarbons, Beijing National Laboratory for Molecular Sciences, College of Chemistry and Molecular Engineering, Peking University, Beijing, 100871, China

[4]Electron Microscopy Laboratory, and International Center for Quantum Materials, School of Physics, Peking University, Beijing, 100871, China

[5]Beijing graphene institute (BGI), Beijing, 100095, China

[6]Academy for Advanced Interdisciplinary Studies, Interdisciplinary Institute of Light-Element Quantum Materials and Research Center for Light-Element Advanced Materials, Peking University, Beijing, 100871, China

[7]State Key Laboratory of Superlattices and Microstructures, Institute of Semiconductors, Chinese Academy of Sciences, Beijing 100083, China

*Correspondence: tbwei@semi.ac.cn; zfliu@pku.edu.cn; jmli@semi.ac.cn; p-gao@pku.edu.cn;

[#]These authors contributed equally to this work.

This supplementary information includes:

Supplementary Figures 1-11 and Tables 1-3

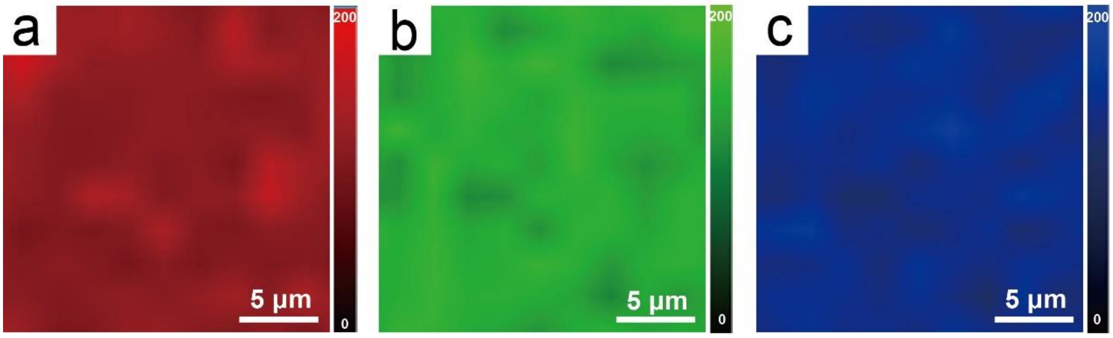

Fig. S1 Raman mapping of Gr covered sapphire substrate. Characteristic Raman peaks mappings of (**a**) D, (**b**) G and (**c**) 2D, respectively.

There are three major Raman peaks of graphene (Gr): D (1341 cm^{-1}) peak, G peak (1585 cm^{-1}) and 2D peak (2684 cm^{-1}). D peak mainly rises due to the presence of disordered sp^3 carbon atoms (Fig. S1a). The G peak originates from the in-plane vibrations of the sp^2 carbon atoms (Fig. S1b). The 2D peak is induced by two phonons double resonance process, thus corresponds closely with the band structure of Gr (Fig. S1c)[1].

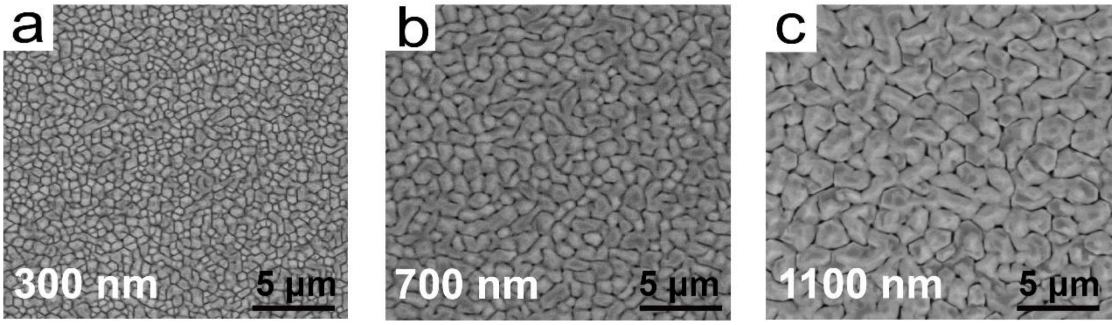

Fig. S2 SEM characterization of AlN films grown on bare sapphire. a-c SEM images of as-grown AlN on bare sapphire with thicknesses of (**a**) 300 nm, (**b**) 700 nm and (**c**) 1100 nm.

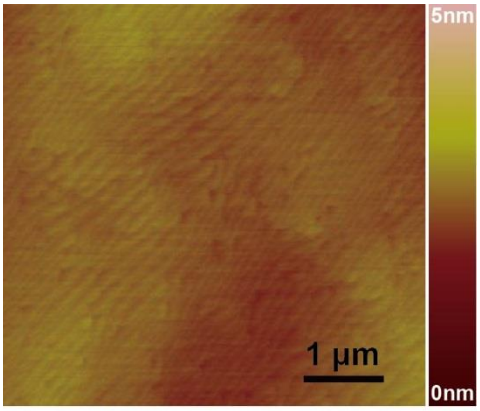

Fig. S3 Atomic force microscope characterization of AlN films grown on sapphire with Gr.

Table S1 Estimated dislocation density of the AlN films with various growth thicknesses.

Thickness (nm) / AlN film (cm^{-2})	50	300	400	500	600	700	1100
Without Gr	7.5×10^{10}	2.54×10^{10}	2.44×10^{10}	2.35×10^{10}	2.29×10^{10}	2.68×10^{10}	7.04×10^{9}
With Gr	6.68×10^{10}	1.02×10^{10}	9.59×10^{9}	8.95×10^{9}	1.17×10^{10}	1.37×10^{10}	2.63×10^{9}

From Table S1, we can see that when the AlN epilayer is only 50nm thick, the dislocation density of the epitaxial layer on Gr is 11% lower than that on bare sapphire. This is because Gr, as the insertion layer, can shield the effect of lattice mismatch between the substrate and the epitaxial layer to some extent[2]. At the same time, even though the AlN film on graphene produces more dislocations than on sapphire during the tensile strain release process, the dislocation density of AlN film on Gr is reduced by 62.6% relative to that on bare sapphire, which is attributed to the role of Gr to shield the substrate and promote dislocation annihilation. This proves that the presence of Gr can effectively reduce the defects in the AlN epilayer.

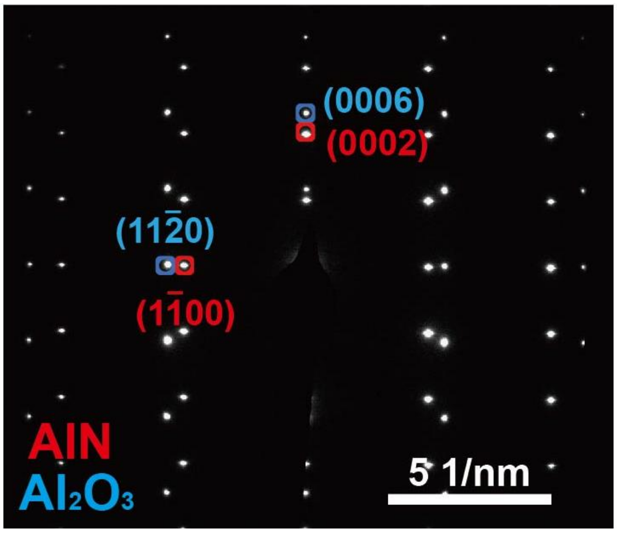

Fig. S4 SAED pattern taken from the interface between AlN and Gr/sapphire.

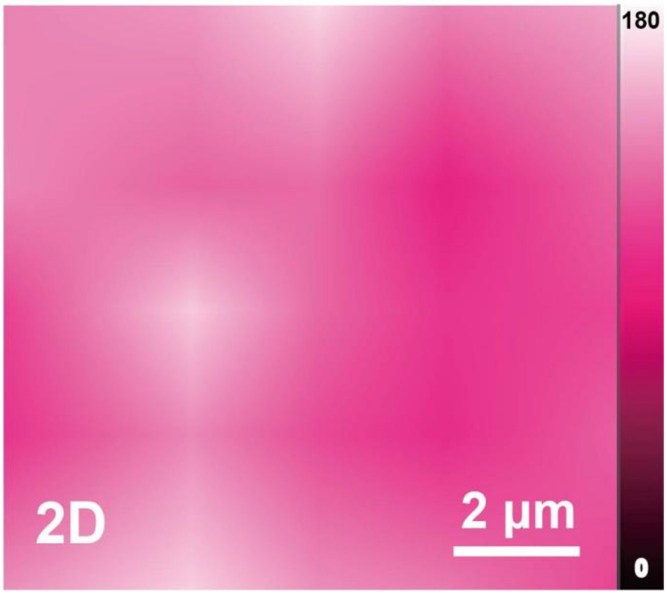

Fig. S5 Raman mapping of a 2D peak on the AlN/Gr/sapphire structure in a 10 × 10 μm^2 region.

The Raman mapping of the 2D characteristic peak in Fig. S5, which is closely corresponding to the band structure of Gr measured on the epitaxial AlN/Gr/sapphire structure[1], confirms the stable existence of carbon element in the form of layered Gr during the whole growth process.

Table S2 E_2 (high) peak position of Raman spectra of AlN films with various growth thicknesses.

Thickness (nm) / AlN film (cm^{-1})	50	300	400	500	600	700	1100
Without Gr	658.5	657.01	656.83	656.65	656.5	658.14	658.02
With Gr	657.23	655.36	655.12	654.98	655.46	657.58	657.49

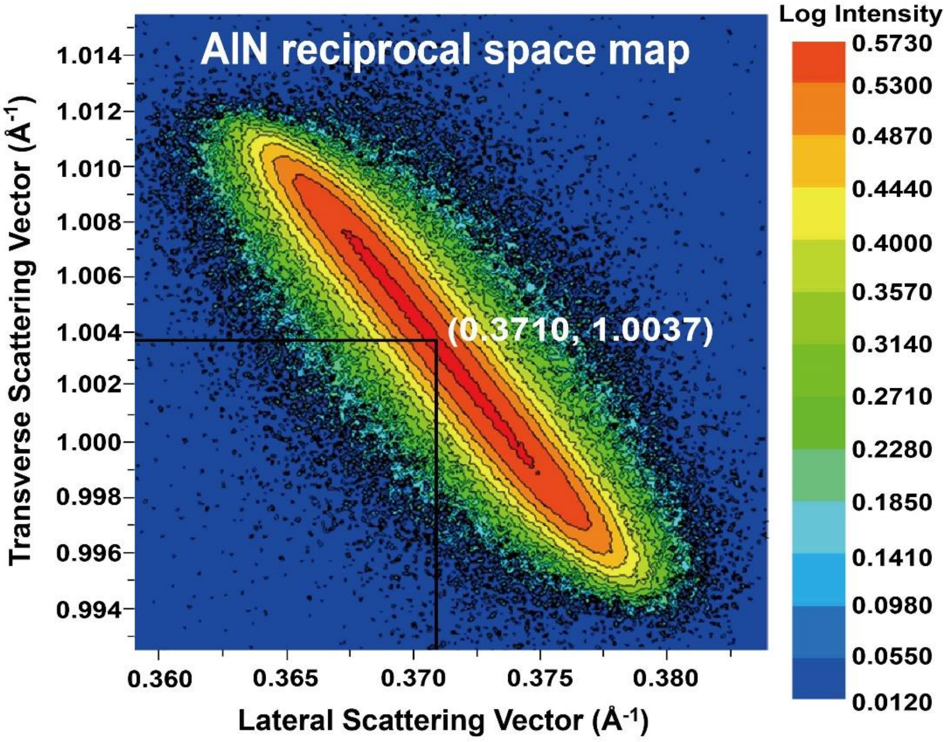

Fig. S6 Reciprocal space maps for the $(10\bar{1}5)$ reflection of the AlN film grown on Gr/sapphire.

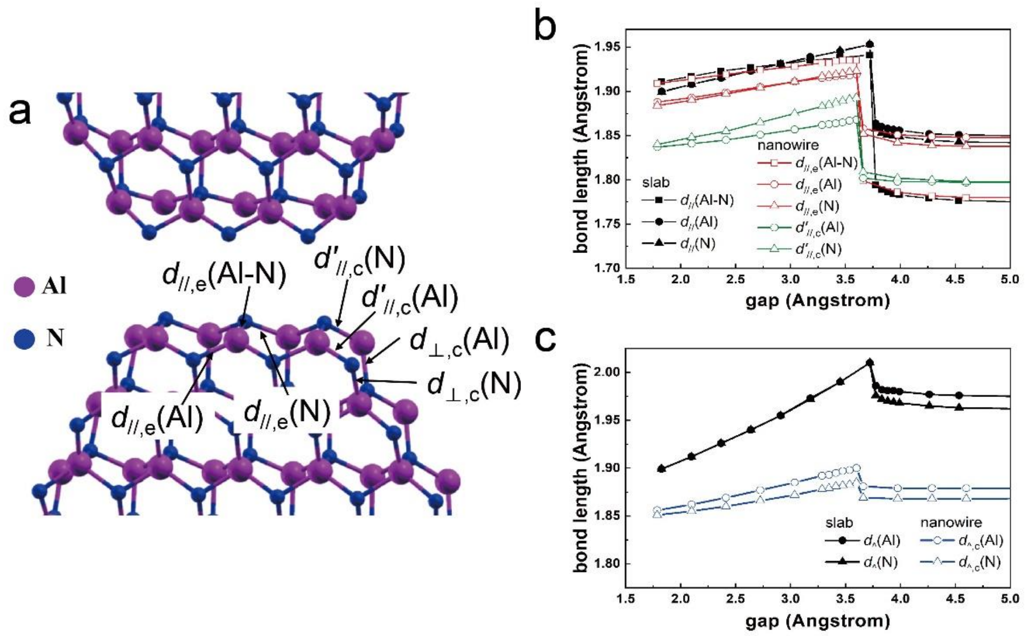

Fig. S7 Density functional theory (DFT) calculations of the other surface bond length at the corner of AlN nanowires during the coalescence. a The other surface bonds of the nanowires. **b** The variation of the parallel bond lengths of nanowires as a function of the separation gap, compared to those bonds of an infinite surface. **c** The variation of the perpendicular bond lengths of nanowires as a function of the separation gap, compared to those bonds of an infinite surface.

Fig. S7 shows the variation of more surface bonds of nanowires during coalescence. As we can see in Fig. S7b, the variation of the parallel surface bonds on the edge of the nanowire follows almost the same trend as those bonds on an infinite surface. This is quite reasonable since these bonds on the edge have a very similar local bonding environment as on an infinite surface. $d'_{//,c}$(Al) and $d'_{//,c}$(N) denote the bond lengths of the Al-N bond at the corner of the nanowire. These two bonds are shared by two adjacent facets and thus have a shorter bond length than the other surface bonds, due to a stronger surface contraction effect. In the small nanowire considered here, the perpendicular bonds at the corner [$d_{\perp,c}$(Al) and $d_{\perp,c}$(N)] are essentially the surface parallel bonds on another facet. Although they are elongated below the critical gap, their bond lengths are much smaller than the other perpendicular bonds. In fact, their bond

lengths are much closer to the parallel bonds around 1.87 Å.

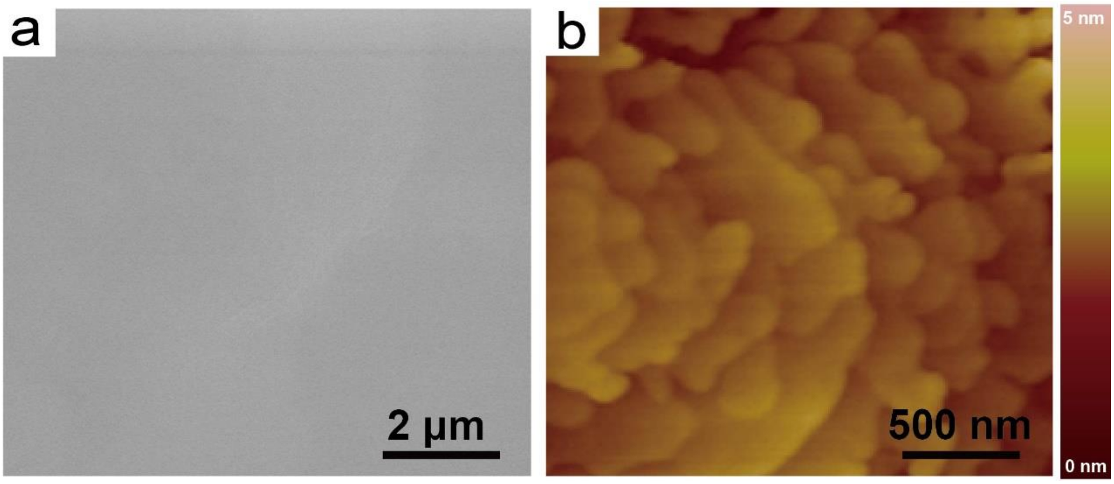

Fig. S8 The morphologies of the DUV-LED structure on Gr/sapphire substrate. a SEM image of as-grown DUV-LED on Gr/sapphire substrate. **b** AFM height image of as-grown DUV-LED on Gr/sapphire substrate.

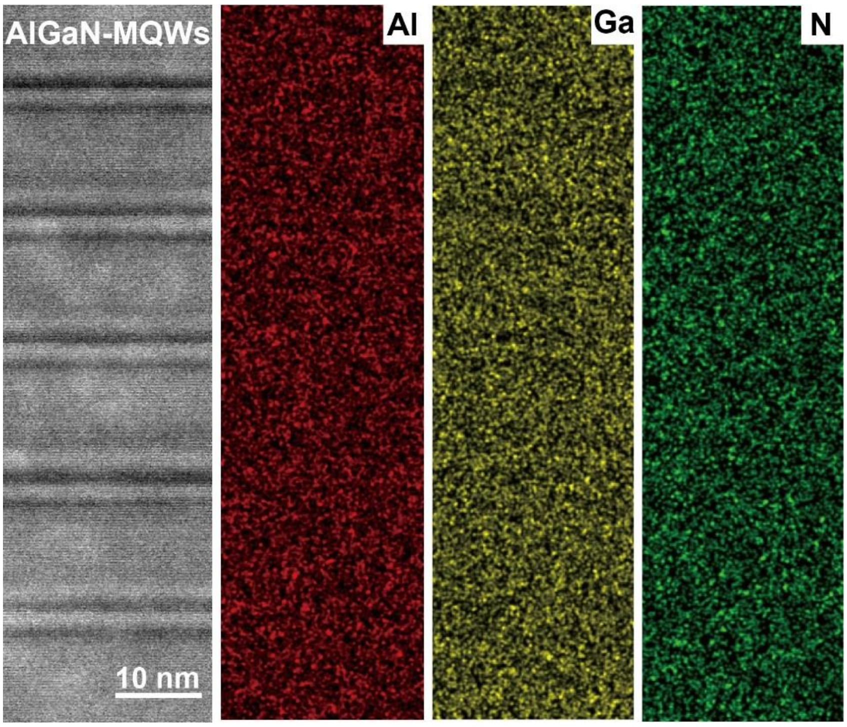

Fig. S9 Cross-sectional STEM image of as-grown DUV-LED with Gr and corresponding Energy dispersive X-ray spectroscopy (EDS) mapping images of Al, Ga and N elements.

Fig. S9 shows the scanning transmission electron microscope (STEM) image of the $Al_{0.4}Ga_{0.6}N/Al_{0.5}Ga_{0.5}N$ multiple quantum wells (MQWs) layers contained in the deep ultraviolet light-emitting diode (DUV-LED) structure, in which the interface between the well and the barrier layer can be clearly observed. At the same time, the AlGaN MQWs structure is characterized by energy dispersive spectroscopy (EDS), and the color distinction due to the change of Ga element composition can be seen in the mapping diagram.

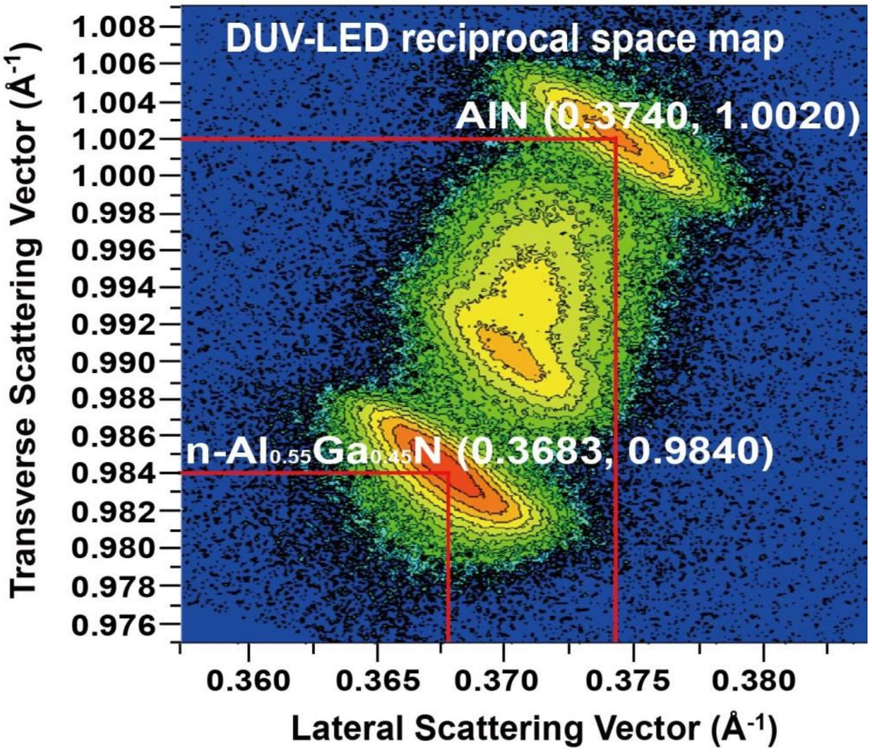

Fig. S10 Reciprocal space maps for the $(10\bar{1}5)$ reflection of the DUV-LED grown on bare sapphire.

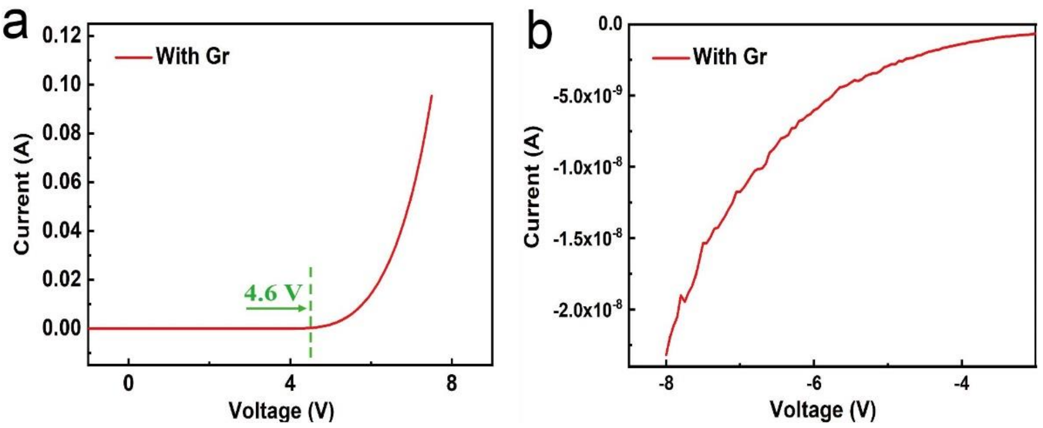

Fig. S11 Current-voltage characteristics of DUV-LED with Gr. a Current-voltage characteristics of as-fabricated DUV-LED. **b** The reverse current-voltage curve of as-fabricated DUV-LED.

Table S3. The corresponding crystal-quality characterization of epitaxial AlN on graphene plasma-treated at different powers.

Power (w)	25	50	100
(0002) FWHM (arcsec)	545	305	506
$(10\bar{1}2)$ FWHM (arcsec)	791	501	794
AlN E_2 (high) (cm^{-1})	657. 90	657.49	657.12

For the heteroepitaxial system of epitaxial AlN on sapphire under the same growth conditions, the compressive strain caused by lattice mismatch and thermal mismatch should be an inherent value. In this work, our experimental route is to control the final growth thickness of AlN film at 1.1 μm (MOCVD growth time is 1 hour), and finally obtain high-quality strain-free AlN film with good surface morphology by optimizing the power of N_2 plasma treatment applied to graphene. In detail, changing the power of N2 plasma applied to graphene can adjust the nucleation density of AlN on graphene. On the one word, and the nucleation island density will affect the crystal quality of AlN epilayer[4]. On the other hand, the size of AlN nucleation island will inevitably decrease (increase) with the increase (decrease) of nucleation density. Therefore, N_2 plasma treatment can effectively adjust the crystal quality and strain state of AlN film grown on graphene.

Reference

1. Zhu, Y. et al. Graphene and Graphene Oxide: Synthesis, Properties, and Applications. *Advanced Materials* **22**, 3906-3924 (2010).
2. Chang, H. et al. Quasi-2D Growth of Aluminum Nitride Film on Graphene for Boosting Deep Ultraviolet Light-Emitting Diodes. *Advanced Science* **7**, 2001272 (2020).
